# Health professional perspectives on lifestyle behaviour change in the paediatric hospital setting: a qualitative study

**DOI:** 10.1186/1471-2431-14-71

**Published:** 2014-03-13

**Authors:** Laura Elwell, Jane Powell, Sharon Wordsworth, Carole Cummins

**Affiliations:** 1 Research and Development, Birmingham Children’s Hospital NHS Foundation Trust, Whittal Street, Birmingham B4 6NH, UK; 2 School of Health and Population Sciences, College of Medical and Dental Sciences, University of Birmingham, Edgbaston, Birmingham B15 2TT, UK; 3 Children and Families Division, Birmingham Community Healthcare NHS Trust, Moseley Hall Hospital, Alcester Road, Birmingham B13 8JL, UK; 4 Joint Commissioning, Coventry City Council, Civic Centre 1, Little Park Street, Coventry CV1 5RS, UK

**Keywords:** Healthy lifestyles, Paediatrics, Health promotion, Qualitative

## Abstract

**Background:**

Research exists examining the challenges of delivering lifestyle behaviour change initiatives in practice. However, at present much of this research has been conducted with primary care health professionals, or in acute adult hospital settings. The purpose of this study was to identify barriers and facilitators associated with implementing routine lifestyle behaviour change brief advice into practice in an acute children’s hospital.

**Methods:**

Thirty-three health professionals (nurses, junior doctors, allied health professionals and clinical support staff) from inpatient and outpatient departments at a UK children’s hospital were interviewed about their attitudes and beliefs towards supporting lifestyle behaviour change in hospital patients and their families. Responses were analysed using thematic framework analysis.

**Results:**

Health professionals identified a range of barriers and facilitators to supporting lifestyle behaviour change in a children’s hospital. These included (1) personal experience of effectiveness, (2) constraints associated with the hospital environment, (3) appropriateness of advice delivery given the patient’s condition and care pathway and (4) job role priorities, and (5) perceived benefits of the advice given. Delivery of lifestyle behaviour change advice was often seen as an educational activity, rather than a behaviour change activity.

**Conclusion:**

Factors underpinning the successful delivery of routine lifestyle behaviour change support must be understood if this is to be implemented effectively in paediatric acute settings. This study reveals key areas where paediatric health professionals may need further support and training to achieve successful implementation.

## Background

Lifestyle behaviour change has great potential to improve child and family health and hence can be considered part of the duty of care of every paediatric health professional. Lifestyle behaviours such as smoking, excessive alcohol consumption, poor diet and lack of physical activity are key contributors to worldwide mortality and morbidity [1-3]. Globally tobacco is the leading threat to public health [4]. Tobacco use often starts during adolescence and according to the World Health Organization an estimated 150 million adolescents currently use tobacco [5]. Passive smoking is also a significant problem, with approximately 700 million children worldwide left vulnerable to the health effects of second-hand smoke exposure [6]. Such health effects include respiratory problems including shortness of breath and exacerbation of asthma, increased incidence of ear infection, and increased risk of sudden infant death syndrome [4]. Evidence indicates that children exposed to passive smoking are at risk of a range of adult onset diseases [7].

In children and young people obesity is a major global problem, with 170 million estimated to be overweight [8]. Health consequences of overweight and obesity include increased risk of lifestyle-related illness including type 2 diabetes, and cardiovascular disease [9]. In addition overweight and obese children suffer psychosocial consequences including social rejection, negative stereotyping, discrimination, body dissatisfaction [10], and reductions in quality of life [11]. The most significant predictor of childhood obesity is parental obesity [12], furthermore obese children are at risk of obesity in adulthood [13].

In the United Kingdom (UK) government public health policy now mandates that health and social care professionals have a responsibility to address lifestyle behaviours such as smoking, poor diet and lack of physical activity, irrespective of healthcare context. This UK initiative is being referred to as ‘Make Every Contact Count (MECC)’ and is being rolled out in England, United Kingdom. Research exists examining the challenges of delivering lifestyle behaviour change initiatives in practice. However, at present much of this research has been conducted with primary care health professionals, or in acute adult hospital settings [14-19]. Little is known about the challenges that acute paediatric health professionals face in relation to delivering lifestyle behaviour change support. This triadic approach to delivering lifestyle behaviour change support may lead to additional challenges for paediatric health professionals compared to those working in adult acute care settings. If lifestyle behaviour change support is to be delivered effectively it is important to consider issues such as the competency and willingness of health professionals to give appropriate healthy lifestyle behavioural advice, as well as consider the healthcare context in which this is to be done. The level of skill and knowledge, and the competencies required by those providing such support will vary according to role and responsibility [20]. Shedding light on practice barriers should facilitate the development of strategies to assist the implementation processes.

We explored the views of paediatric health professionals on supporting lifestyle behaviour change with hospital patients and their families through a qualitative study. The research was carried out in a paediatric hospital setting in the UK where lifestyle behaviour change advice has been broadly defined to include brief contacts with patients aged over twelve years, as well as contacts with all families. Brief contacts include activities such as advice giving and directing to other support services, raising awareness of risks, or providing encouragement or support for lifestyle change. It is suggested that these activities range from 30 seconds in duration to a couple of minutes [21].

## Methods

### Design and setting

Thirty three face to face semi-structured interviews were conducted with clinical staff (nurses, junior doctors, allied health professionals and clinical support staff) from inpatient and outpatient services provided at Birmingham Children’s Hospital, United Kingdom, a hospital providing acute secondary and tertiary care to children and young people. Interviews were conducted by the first author. A qualitative semi-structured interview design was chosen to allow useful exploration of attitudes and beliefs towards content of interest. This study was defined as service evaluation by the National Research Ethics Service and therefore NHS Research Ethics Committee approval was not needed.

### Sample and recruitment

Participants were purposively sampled to incorporate a range of hospital staff with patient contact including; medical specialities and support staff such as housekeepers and healthcare assistants. Job roles and levels of training in relation to providing brief lifestyle behaviour change advice were also considered during sampling. The hospital health promotion lead (JP) provided contact details of managers for hospital inpatient and outpatient departments. A researcher (LE) then arranged interview sessions at a convenient time dependent on clinical workload. The researcher re-booked sessions if necessary to ensure different job roles and training levels were incorporated within the sample. The majority of participants worked across inpatient and outpatient services.

### Data collection

The interviews were conducted during February and March 2012 and lasted approximately 19 minutes (standard deviation 7 minutes). Participants were approached in person within their department whereby a researcher explained the study aims and provided a participant information sheet. Interviews took place until data saturation was reached. During the period of data collection health professional training was taking place in the hospital in relation to supporting lifestyle behaviour change assessment and support, hence some but not all participants had received training.

A semi-structured topic guide (see the ‘interview topic guide’ section) was used throughout the interviews. Interview questions were generated through discussions with the research team and health promotion leads at the hospital. The main focus of the questions was to understand health professional feelings towards the MECC initiative and delivering brief lifestyle behaviour change advice, which for the purpose of this study was defined in relation to smoking and obesity-related behaviours as these were a priority focus for the hospital. Questions relating to current knowledge and skills, as well as beliefs in relation to responsibilities, were explored during the interviews.

### Interview topic guide

• Current level of lifestyle behaviour change knowledge and skills

• Beliefs about responsibilities for addressing lifestyle behaviour change

• General attitudes towards MECC and brief opportunistic advice

• Attitudes towards lifestyle behaviour change and hospital healthcare context

• Training issues and resources and addressing lifestyle behaviour change

• Reflections on prior experiences of addressing lifestyle behaviour change

### Data analysis

Interview findings were analysed using a thematic framework analytical approach. Thematic framework analysis was chosen as it is considered appropriate for policy-related applied research that has short timescales [22]. This involved an iterative process of transcribing the interviews, re-familiarisation with interview content, systematically open coding interview content including consideration of conflicting data, producing a coding framework and then re-coding interview content in line with the framework. Following coding of all interviews content codes were collated into key themes. Coded participant data was then charted into a matrix for each theme, mapping and interpretation followed this stage where associations between and within participants and themes were made. To enhance reliability, data was independently coded by additional researchers (LE, CC, SW). Qualitative analysis software was used to support the analytical process (NVivo version 9.2).

## Results

Thirty three members of staff were recruited. This included nursing staff (*n = 22*), junior doctors (*n = 2*), clinical support staff (*n = 6*) and allied health professionals (*n = 3*). The sample of staff who took part in the interviews was predominantly female (91%). The median age was 29.7 (range 18–55). The average length of time that staff interviewed had been in their profession was 10 years and 1 month (range 3 months to 34 years). The average length of time that staff interviewed had worked for Birmingham Children’s Hospital was 6 years and 10 months (range one month to 24 years). Out of the ten participants interviewed from wards offering the MECC training during the period in which interviews were being conducted, six had completed the training.

Three master themes emerged from the data: ‘paediatric hospital environment’, ‘health professional knowledge, beliefs and behaviours’ and ‘patient and family related challenges’. Here we focus on one main theme, the ‘paediatric hospital environment’. This theme covers the challenges of delivering brief lifestyle advice in the paediatric hospital setting and incorporates five sub-themes*; ‘experience of effectiveness’, ‘capacity constraints’, ‘the ‘right’ time’, ‘anticipated benefits’,* and *‘staff support resources’.* The other themes cover material less specific to the paediatric hospital setting, ‘health professional knowledge, beliefs and behaviours’, and ‘patient and family related challenges’ are reported elsewhere.

### Experience of effectiveness

Participants felt that there was little visible evidence available to them to demonstrate the effectiveness of providing lifestyle change brief advice in this setting. This perspective stemmed from the uncertainty as to whether they would come into contact with the same patient and family again in the future;

“*well yeah, I mean our patients they you know, we get them home as soon as possible so we don’t get to see the results”* (Nurse 11, 9 years in profession, not MECC trained).

This lack of evidence may contribute to disengagement with supporting lifestyle change, particularly if a conversation with a patient or family about lifestyle change has proved challenging previously;

*“if you can’t see the benefits of what you are doing it’s really hard to keep engaging with it”* (Doctor 24, 6 months in profession, not MECC trained).

In contrast when participants had witnessed families having made changes to their lifestyles, offering support felt worthwhile. Although at the same time it was acknowledged that for some paediatric sub-specialties such opportunities rarely arise;

*“we notice some changes with them and that’s the rewarding bit then, is that you get some feedback and I think not all ward areas are that lucky that they’ve got the same people coming in and out”* (Nurse 26, 15 years in profession, not MECC trained).

### Capacity constraints

Time constraints were frequently mentioned as a factor that determined whether lifestyle change conversations took place. For example one participant emphasised concern about conflicting priorities:

*“I'm normally all over the place doing like five, six different things so I think it's, this isn't always, on my top of priorities”* (Support Staff 16, 1 year in profession, MECC trained)

Traditional nursing care duties were regarded as a greater priority, particularly when patients were admitted for difficult medical conditions and therefore may require more clinical attention;

*“the patients we get through are very complex so it’s not always something that comes to the front of your mind when you’re doing your medicines”* (Nurse 20, 5 years in profession, MECC trained)

A further challenge was that it was difficult to predict how long the conversation about lifestyle change would take, especially if the patient or family were interested in discussing this during a busy shift;

*“It could be a discussion that you end up being there for sort of an hour with, couldn’t you, and you just don’t know which way it’s going to go”* (Nurse 21, 29 years in profession, MECC trained)

As a consequence this may lead to situations where it is easier not to instigate conversations in order to protect time needed for clinical duties;

*“people won’t wanna ask in case then that parent goes, ‘well what can I do and what can I do here’ and it’s half hour of your time gone if they ask that question potentially”* (Nurse 20, 5 years in profession, MECC trained)

The hospital environment was also at times a barrier to engaging in conversations about lifestyle change. For example, it was felt that privacy was an issue, especially in relation to discussing lifestyle topics that may be perceived as sensitive, for instance talking about sexual health with young people;

*“we’ve got a four bedded bay area so conversations in there are difficult”* (Nurse 26, 15 years in profession, not MECC trained)

Similarly, participants felt unable to display some public health information aimed at teenagers when they knew that the environment was shared by younger children due to joint outpatient clinic schedules;

*“I think having mixed clinics, paediatrics and adolescents clinics together um doesn’t give the opportunity for health promotion to be…so you probably wouldn’t want lots of posters and information about smoking and alcohol and drugs and sex if you’ve got small children around”* (Allied Health Professional 8, 6.5 years in profession, not MECC trained)

Continuity of information was an area of concern in that patients could receive different information depending on who was delivering lifestyle change support. For example, one participant discussed the issue of different health care workers providing contrasting information and emphasised the need to be ‘singing from the same sheet’;

*“I think you know doctors will give slightly different information to nurses, who will give slightly different information to occupational therapists and dieticians, and everyone’s got their bit that they know more about, and a different way of delivering it, and you know sometimes people will relate more to one than they will to the other so I think, but I think the main thing is people have to be like singing off the same sheet so to speak, so they are all giving a consistent message, whether it is delivered slightly differently they are all giving the same message.* (Doctor 24, 6 months in profession, not MECC trained)”

Another member of staff was unsure whether healthy lifestyle messages delivered in the hospital setting would be reinforced in the community setting;

*“continuity I suppose is a big challenge, of whether that’s going to carry on in the community setting”* (Nurse 4, 7.5 years in profession, not MECC trained)

### The ‘right’ time

The question was raised as to whether it was appropriate to discuss lifestyle change at a time when families are under pressure due to having a sick child admitted into the hospital;

*“in six months time could you be holding their hand whilst their child dies? And all you’d be thinking of is ‘oh my god I told him he was too fat six months ago and he needed to lose a bit of weight’”* (Nurse 15, 34 years in profession, not MECC trained)

In contrast one participant perceived the children’s hospital setting as an appropriate way to reach patients that may infrequently come into contact with healthcare services or health professionals;

*“I feel very strongly that it’s the ideal setting really…because they’re here for health reasons a lot of young people won’t go to the GP without a parent in tow or just wouldn’t go at all, so I think we’re ideally placed to be able to give more support”* (Nurse 26, 15 years in profession, not MECC trained)

Uncertainly also existed in relation to the timing of a lifestyle change conversation and the point at which these issues should be raised during a longer hospital stay;

*“I think it gets missed a lot here because we haven’t got an appropriate time to ask it on admission and if it’s in the middle of the night, you don’t find it’s an appropriate time to be asking them questions as well”* (Nurse 22, 5 years in profession, not MECC trained)

### Anticipated benefits

The benefits that could potentially arise from providing healthy lifestyle advice were an incentive for health workers to engage in providing lifestyle change support.

Benefits for the organisation and NHS as a whole including cost savings were mentioned;and reductions in hospital admissions;

*“We have to reduce the cost on the NHS at the moment. If you look at the global picture of the NHS, you know, we’ve got to save a lot of money and health promotion is one of those ways”* (Nurse 1, 20 years in profession, not MECC trained)

*“often a health promotion message could prevent future admissions not just on the mental health side but also on the medical and potentially the surgical side”* (Nurse 30, 30 years in profession, not MECC trained)

Having conversations with families about lifestyle change to promote health were found to be professionally and personally rewarding;

*“part of the job satisfaction is knowing that you’ve done something to help somebody”* (Nurse 11, 9 years in profession, not MECC trained)

Benefits of providing brief advice were also discussed in terms of how this presents an opportunity to affect change early on before unhealthy lifestyle behaviours become a permanent factor in the lives of children and young people;

*“it’s important for us because we’re accessing young people when their personalities and their behavioural traits aren’t fully formed so we’ve got a much better opportunity to change future behaviours to impact on long term health”* (Nurse 30, 30 years in profession, not MECC trained)

Furthermore it presents an opportunity to impact on the lives of children and young people through influencing the behaviour and lifestyles of parents and guardians;

*“if you keep the parents healthy that will help the children in the long run”* (Nurse 20, 5 years in profession, MECC trained)

### Health professional support resources

Lifestyle change support was viewed as a health education activity, in the sense that providing patients and families with knowledge as to why they should change should in turn lead to behaviour change;

*“Explaining to um patients and also staff um the benefits of certain lifestyle choices um in terms of, like eating healthy, exercising and things like that, and also the disadvantages of doing other things, drinking, smoking, excess weight, and trying to educate them in a way that makes them understand why certain things are good and certain things are bad to change their behaviour”* (Doctor 24, 6 months in profession, not MECC trained )

However one participant also acknowledged that health education alone isn’t always sufficient to lead to a change in behaviour;

*“Health promotion I think needs to get that across that it’s not just about providing the right and correct healthy lifestyle, but actually about why people may choose different options or why people would refuse to take that advice”* (Allied Health Professional 8, 6.5 years in profession, not MECC trained)

Access to health promotion resources was a problem at times and health professionals reported that resources such as leaflets were often not available when an opportunity to intervene presented;

*“I personally find that the leaflets aren’t available when you actually need them” (*Allied Health Professional 8, 6.5 years in profession, not MECC trained*)*

Participants also reported that it would be helpful to have more access to resources to facilitate health promotion activity within the hospital;

*“I think it would be useful to have more info that you could give them for them to read at their leisure”* (Nurse 25, 3.5 years in profession, MECC trained)

The effectiveness of written resources such as leaflets was also discussed, with mixed feelings. It was felt that only motivated people would access resources. One participant provided an account of their own experiences of not wishing to access health promoting material, and contrasted this with the notion of providing resources to young people;

*“I don’t know because looking back to when I was a teenager if I was given a leaflet would I read it? Or would I just look at it”* (Nurse 12, 1 year 9 months in profession, not MECC trained)

Nevertheless leaflets could be helpful in situations where families may feel self-conscious about asking for support;

*“sometimes people might be a little bit embarrassed about asking questions you know. Or, we’ve had parents that think that you will think that they’re a bad parent because they’re asking certain things”* (Nurse 17, 10 years in profession, not MECC trained)

It was also suggested that they shouldn’t replace verbal information, but could prove a useful tool for staff, for example in supporting healthy lifestyle conversations;

*“I think if you make a point of explaining it alongside, I think if you just give out a leaflet people don’t necessarily, but if you kinda look at it with them”* (Nurse 4, 7.5 years in profession, not MECC trained)

Requests were made for information packs for health professionals that contained up to date guidance in relation to key health promotion topics. It was felt that this could further support staff in engaging in lifestyle change conversations;

*“I don’t know if we have something on it or not, but if we had something that had the latest guidelines and articles, that people could just dip in and out of and see, that would be really helpful and if there was some way you could find it easily”* (Doctor 24, 6 months in profession, not MECC trained)

## Discussion

We have for the first time identified a range of barriers, as well as facilitators in relation to health workers with patient contact delivering healthy lifestyle behaviour change advice to children, young people and their families in hospital. Barriers included a lack of feedback to demonstrate effectiveness and capacity constraints relating to time and the hospital environment. Facilitators included perceived benefits that could result from lifestyle behaviour change advice, such as cost savings and reduced admissions. In general, hospital health promotion was viewed as a health education activity.

Participants showed concern that there was infrequent opportunity to receive feedback about the outcomes of lifestyle behaviour change advice previously provided. These findings suggest that the provision of feedback from patients and families or community services to acute health professionals may reassure them that their efforts are worthwhile. Despite this concern, the interviews also revealed that some health professionals perceived benefits could arise, such as reduced hospital admissions and cost-savings, and this was an incentive to supporting a public health focus in hospitals. Furthermore, health professionals also mentioned that providing lifestyle behaviour change advice presents a chance to make a difference to a child’s well-being through intervention with the family, which was viewed as personally and professionally rewarding. This finding further supports the recommendation that feedback to acute health professionals about what difference their input has made to the family could be beneficial in reinforcing health professional engagement with public health initiatives.

Our findings echo conclusions from a recent government enquiry undertaken in the United Kingdom to understand the role that behaviour change research plays in the formulation of policy. It concluded that there is a lack of applied research at population level to support specific interventions to change the behaviour of large groups [5]. Evidence to suggest that brief lifestyle change advice is effective in paediatric hospital settings is scarce.

Health professionals were concerned that talking about lifestyle behaviours my lead into longer conversations that could deter from clinical duties. In addition more complex lifestyle change conversations may result from initial enquiries. Health professionals may not feel comfortable engaging in these conversations, as confidence was also a factor identified as a barrier. In contrast brief lifestyle behaviour change advice is defined by policy makers as quick to deliver. Evidence from another qualitative study conducted with ward nurses [23] has reported similar findings in relation to time constraints, whereby health promotion activity was viewed as an optional extra following the ‘real work’ of nursing duties being completed. This has been echoed in other studies [24-26].

The interviews revealed that health professionals working in a children’s hospital view health promotion as an educational activity which aims to increase knowledge in order to change behaviour. This may explain why health professionals were concerned with the availability of health promotion resources to assist healthy lifestyle discussion. Whilst behaviour change guidance in the UK has acknowledged the role of education [20] other research has argued that health professionals should avoid the view that knowledge and provision of health promotional materials will lead patients to change their behaviour [27]. Therefore if lifestyle behaviour change initiatives are to be implemented successfully we need to further understand and address health professional training needs [21].

### Strengths and limitations

A strength of this study is that it is one of the first to provide information on the barriers to implementation of lifestyle behaviour change routine advice in a hospital setting. Furthermore the interview findings were independently analysed by three researchers, enhancing creditability.

A limitation of the study is that results may not generalise to all health professional groups with patient contact within hospitals. In addition, the interviewer’s position as a researcher within the hospital, although not known to the respondents, may have influenced the participant views expressed and our analysis should be read taking this into account.

Some of the interviews we conducted were relatively short and therefore we considered whether researcher-participant interaction was at risk of being constrained by social desirability. However, analysis of even the short interviews revealed that participants discussed both positive and negative views in relation to providing lifestyle behaviour change advice, presenting a rich data set.

In addition MECC was already in the process of being piloted on four general medical wards at the hospital during the research, which may have impacted on participant views. Alternatively these early experiences of trialling MECC in this setting may have merely stimulated participant opinion.

## Conclusion

Health professional support for lifestyle behaviour change may be viewed as an essential element of professional practice in children’s hospitals and other settings with great potential to improve child and family health outcomes. We have described factors influencing whether health professional delivery of routine lifestyle behaviour change support will be implemented effectively in the paediatric hospital setting. It is important to understand these factors prior to embedding such initiatives, if they are to be successful. This study has revealed that in the paediatric hospital setting health professionals recognise the benefits that can result from delivering lifestyle behaviour change advice. We recommend, however, that systems are put in place to provide feedback to individual health professionals in relation to outcomes of support given to children, young people and their families and to promote potential benefits to all health professionals. We also recommend that health professional support and training is provided to ensure that public health initiatives are not delivered solely as health education activities within acute settings. We are therefore now developing training that incorporates real life examples of advice leading to behaviour change.

## Consent

Written informed consent was obtained from the participants in relation to publication of this report.

## Abbreviations

MECC: Make Every Contact Count.

## Competing interests

The authors declare that they have no competing interests.

## Authors’ contributions

LE, JP, and CC conceptualised and designed the study design. LE and JP managed recruitment. LE conducted the interviews. LE, SW, CC analysed the data. LE developed the paper with contribution from SW, CC. All authors approved the final manuscript submitted.

## Pre-publication history

The pre-publication history for this paper can be accessed here:

http://www.biomedcentral.com/1471-2431/14/71/prepub
